# Microbiome Composition in a Common Mediterranean Bryozoan Following an Unprecedented Marine Heatwave

**DOI:** 10.1111/1758-2229.70185

**Published:** 2025-09-04

**Authors:** Blanca Figuerola, Cristina Linares, Claudia Aparicio‐Estalella, Paula López‐Sendino, Joaquim Garrabou, Javier del Campo

**Affiliations:** ^1^ Departament de Biologia Marina i Oceanografia Institut de Ciències del Mar (ICM‐CSIC) Barcelona Spain; ^2^ Departament de Biologia Evolutiva Ecologia i Ciències Ambientals, Facultat de Biologia, Universitat de Barcelona (UB) Barcelona Spain; ^3^ Institut de Recerca de Biodiversitat (IRBio) Barcelona Spain; ^4^ Institut de Biologia Evolutiva (CSIC–Universitat Pompeu Fabra) Barcelona Spain

**Keywords:** bryozoans, marine calcifiers, marine heatwaves, microbiome, thermal stress

## Abstract

Marine heatwaves are intensifying due to global warming and increasingly drive mass mortality events in shallow benthic ecosystems. Marine invertebrates host diverse microbial communities that contribute to their health and resilience, yet microbiome responses under thermal stress remain poorly characterised across most taxa. Here, we characterise the microbiome composition in colonies of the common Mediterranean bryozoan *Myriapora truncata* at two depths (13 and 17 m) following the extreme 2022 marine heatwave. Despite no visible necrosis, microbial communities at both depths exhibited shifts indicative of thermal stress, including the reduced presence of potential core microbial members. Colonies from the shallower, warmer depth showed higher alpha diversity and reduced abundance of key functional genera compared to deeper colonies, suggesting early dysbiosis. These results highlight that *M. truncata* —though visually unaffected—undergoes sublethal microbiome alterations under thermal stress. This study provides the first characterisation of a bryozoan microbiome after a marine heatwave and highlights the potential of host‐associated microbial communities as early bioindicators of invertebrate stress in a warming ocean.

## Graphical abstract

*Myriapora truncata* exhibits microbiome compositional changes after the 2022 marine heatwave, despite the absence of visible necrosis. These findings highlight host‐associated microbes as early indicators of thermal stress in Mediterranean benthic ecosystems.

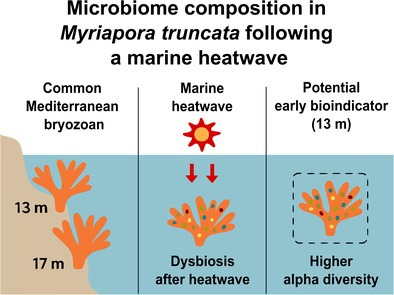

## 1 Introduction

Marine heatwaves (MHWs)—defined as prolonged periods of abnormally high sea surface temperatures—are increasing in frequency, intensity, spatial extent, and duration due to global warming (Frölicher et al. 2018). The Mediterranean Sea, characterised by its semi‐enclosed and relatively shallow nature, is particularly vulnerable, warming approximately three times faster than the global average, a trend directly linked to the rising frequency of MHWs (IPCC et al. 2019; Lejeusne et al. 2010). Over the past decade, the Western Mediterranean Basin has experienced an increase in MHW frequency, leading to mass mortality events among benthic species (Garrabou et al. 2022).

The summer of 2022 marked one of the most extreme MHWs recorded in the NW Mediterranean Sea (Guinaldo et al. 2023), with up to 50 days of anomalous temperatures (www.t‐mednet.org). This extreme event caused unprecedented mortality in the octocoral *Paramuricea clavata* , a key structural species in benthic communities (Estaque et al. 2023; Rovira et al. 2024). However, its MHW‐induced mortality varied across spatial and temporal scales (Estaque et al. 2023; Rovira et al. 2024), as previously observed in this and other species following MHWs (Garrabou et al. 2022). While mortality incidence has been significantly linked to the number of MHW days reported, high variability remains in the extent of mortality impact under similar thermal exposure (Garrabou et al. 2022). This variability may be attributed to a combination of factors, including thermal exposure duration and intensity (Elzahaby et al. 2021), local hydrodynamics (Lenihan et al. 2008), species‐specific thermal thresholds (Gómez‐Gras et al. 2019) and ecological memory shaped by prior exposure to MHWs—which may play a crucial role in modulating the physiological and adaptive responses of populations to future MHWs (Hackerott et al. 2021; Hughes et al. 2019).

The mechanisms underlying memory responses in marine invertebrates are increasingly recognised as being mediated not only by the host but also by the associated microbiome (the holobiont), which includes bacteria, archaea, microbial eukaryotes, and viruses (Hackerott et al. 2021; Vompe et al. 2024). The microbial communities contribute to host health through nutrient recycling, the production of defensive compounds, and other beneficial functions (van de Water et al. 2018). Due to their short generation cycles, large population sizes, and diverse metabolic capabilities, microbes can rapidly respond to environmental changes, thereby enhancing holobiont plasticity and thermal stress adaptation, as demonstrated in corals (Ziegler et al. 2019). Conversely, thermal stress can disrupt holobiont balance, leading to shifts in microbial composition that favour opportunistic pathogens while diminishing beneficial symbionts, ultimately compromising host resilience (Corinaldesi et al. 2022).

Characterising microbiome composition and its potential functional role under varying thermal conditions can offer valuable insights into holobiont capacity for acclimatisation, resilience, or resistance to these increasing MHWs (Bonacolta et al. 2024; Corinaldesi et al. 2022; Prioux et al. 2023). However, our current understanding is limited by the taxonomic biases in marine invertebrate microbiome studies, which have primarily focused on a few phyla, such as Cnidaria (e.g., corals) and Porifera, while largely overlooking others (Boscaro et al. 2022). One such underrepresented phylum is Bryozoa (Boscaro et al. 2022; Bourne et al. 2013; Figuerola et al. 2025; Li et al. 2019; Patin et al. 2019); despite its global abundance and diversity, its ecological importance—particularly in habitats such as Mediterranean coralligenous assemblages (Ballesteros 2006), where new taxa and diagnostic traits continue to be discovered (Rosso et al. 2025)—and its potential as a sensitive model holobiont for studying the biological and mineralogical impacts of global change (Figuerola et al. 2023, 2025; Smith 2009). Bryozoa is the second most impacted phylum by MHWs in the Mediterranean Sea, following Cnidaria (Garrabou et al. 2022). However, few studies have specifically assessed the impact of heatwaves on their populations (Pagès‐Escolà et al. 2018). Given that some bryozoan species are considered relevant habitat‐forming organisms that enhance structural complexity and support biodiversity (Cocito 2004), their decline could thus lead to significant shifts in these assemblages, potentially compromising ecosystem resilience. Despite the suggested role of the bryozoan microbiome in enhancing host resilience to environmental changes and serving as an early indicator of health deterioration (Figuerola et al. 2025), no studies to date have characterised bryozoan‐associated microbial communities following marine heatwave events.

This study examines the microbiome (i.e., the bacterial taxonomic composition) of the common Mediterranean bryozoan *Myriapora truncata* following the extreme 2022 MHW event. Previous research combining field surveys and thermal stress experiments has shown that *M. truncata* demonstrates higher thermal tolerance than the sympatric bryozoan *Pentapora fascialis*, with greater survival and fewer visible signs of necrosis under elevated temperatures (Pagès‐Escolà et al. 2018). Although no exact thermal threshold has been established, *M. truncata* has survived sustained exposures up to 27°C without exhibiting lethal effects, unlike *P. fascialis*, which exhibited pronounced mortality at lower temperatures. Long‐term monitoring also documented sharp declines in *P. fascialis* colony cover following marine heatwaves, with up to 86% reduction observed after the 1999 warming event (Cocito and Sgorbini 2014). Additionally, field studies during MHWs reported limited or no visible mortality in *M. truncata* colonies (Rovira et al. 2024), though sublethal signs, such as epiphytized skeletons, were occasionally observed. These findings suggest *M. truncata* may be relatively resistant to acute temperature anomalies in terms of survival, but sublethal impacts, particularly those affecting the host‐associated microbiome, remain largely unexplored. To address this gap, we first characterised the microbiome composition of colonies collected from two depths, both of which were previously exposed to the MHW but showed no signs of necrosis, which supports the high species resistance observed in previous studies. We then compare colonies at 17 m, where lower temperatures likely preserved a state closer to pre‐MHW conditions, with those at 13 m, which experienced greater thermal stress and may exhibit early microbiome dysbiosis. Under this framework, we consider deeper colonies to be a reference for a ‘healthy’ baseline and shallower colonies to be potentially ‘dysbiotic’ due to the lasting effects of the MHW. We hypothesise that these apparently healthy colonies will maintain relatively stable microbial communities across depths, supported by the capacity of host‐associated microbial communities to acclimatise and retain memory of past MHW exposures (Hackerott et al. 2021), as well as *M. truncata* 's resilience to certain levels of thermal stress (Pagès‐Escolà et al. 2018). However, given the unprecedented intensity of the 2022 MHW (Estaque et al. 2023), we also anticipate subtle but detectable microbial changes in the shallow colonies, potentially serving as early indicators of host stress or incipient dysbiosis.

## 2 Materials and Methods

### 2.1 Study Area

The study was conducted in Pota del Llop site (42° 2′ 58.920″ N, 3° 13′ 31.440″ E) at the Medes Islands Marine Reserve, located within the Montgrí, Illes Medes and Baix Ter Natural Park (NW Mediterranean Sea). In September 2022, six bryozoan colonies were collected, with three replicate colonies sampled at each of two depths (13 and 17 m). These depths were selected based on observations of mass mortality in the octocoral *P. clavata* following the 2022 MHW event in some sites of the Medes Islands (Rovira et al. 2024). Unlike *P. clavata* , *M. truncata* did not show a significant increase in mortality at these sites; though many colonies exhibited epiphytized skeletons, indicating old mortality from previous years (Linares et al. 2005; Rovira et al. 2023).

While the 13 and 17 m depths are relatively close, long‐term temperature records (2018–2022) show consistent differences between them, suggesting vertical thermal structuring rather than variability driven by isolated meteorological events (see Figure S1). This pattern is consistent with broader regional trends reported by Garrabou et al. (2022), where the strongest warming over the 2010–2019 period occurred in shallower layers (e.g., 10 m), with a progressive attenuation of warming with depth. Moreover, subsurface MHW frequency has been shown to decline with depth—averaging 73 days at the surface vs. 47 ± 25 days at 20 m—highlighting how even modest vertical gradients can reflect meaningful differences in thermal exposure.

### 2.2 Temperature Time Series

**FIGURE 1:**
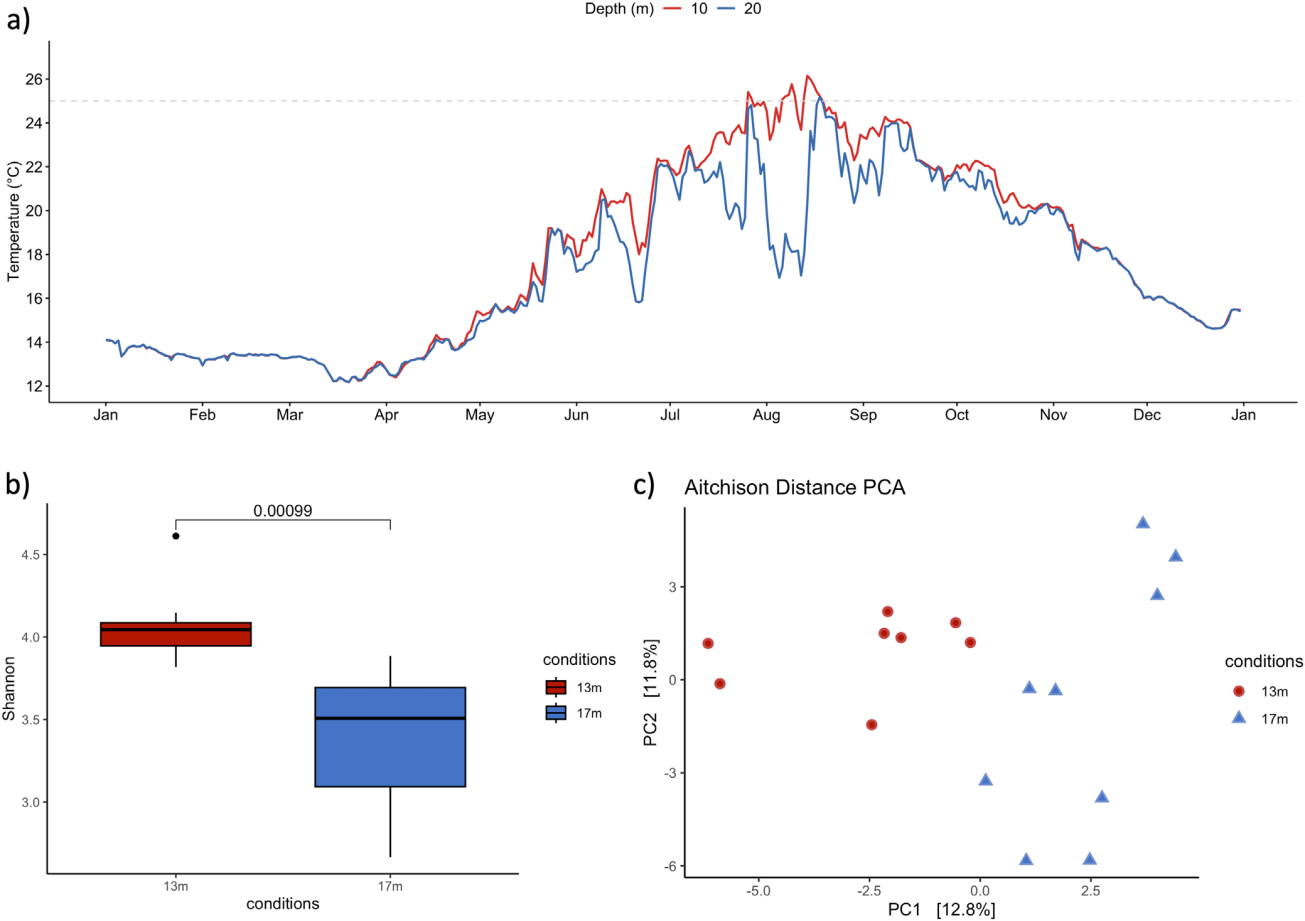
Daily mean seawater temperature in 2022 (a), and alpha‐ (b) and beta‐diversity (c) of the microbiome of *Myriapora truncata* at the studied depths in Pota del Llop. Daily mean temperature was calculated from hourly measurements recorded by in situ hobo sensors at 10 and 20 m depth in 2022. The alpha diversity of bacterial communities differed significantly between both depths (two‐sample Kolmogorov–Smirnov test; *p* < 0.05). Beta diversity also showed significant differences between depths (ANOSIM; *p* = 0.019).

Seawater temperature data from 10 and 20 m depths in Pota del Llop in 2022 were obtained from the T‐MEDNet platform (http://www.t‐mednet.org/). Temperature data loggers (HOBO TidbiT v2, Onset) recorded hourly measurements, from which daily averages were obtained (Figure 1a).

### 2.3 Microbiome

#### 2.3.1 DNA Extraction

After collection, bryozoan colonies were transported on ice in seawater‐filled plastic bags. In the laboratory, colonies were rinsed in milliQ water, preserved in 75% ethanol, and stored at −20°C until DNA extraction. From each colony, three subsamples (~1 mm^2^) were cut from the growing edge, yielding 18 subsamples in total. These subsamples were transferred into a 1.5‐mL Eppendorf tube. DNA was extracted from each subsample using the DNeasy Blood & Tissue Kit (Qiagen, Hilden, Germany), following the manufacturer's protocol, and stored at −20°C until sequencing.

#### 2.3.2 16S rRNA Gene PCR Amplification and Sequencing

DNA concentrations were measured with a Qubit fluorometer, and all subsamples yielded a concentration above 1 ng/μl. Subsamples were sent to the Integrated Microbiome Resource facility (Dalhousie University, Canada) for amplification and sequencing of the V4 region of the prokaryotic 16S rRNA gene using the modified primers 515F (GTGYCAGCMGCCGCGGTAA) and 806RB (GGACTACNVGGGTWTCTAAT) (Apprill et al. 2015; Parada et al. 2016). Sequencing was performed on the Illumina MiSeq platform. This approach allowed characterisation of the bacterial taxonomic composition (microbiome) associated with the samples.

#### 2.3.3 Bioinformatics and Statistics

Raw sequencing data are available in NCBI (BioProject: PRJNA1236962). Primers and adaptors used for sequencing were removed from paired‐end reads using Cutadapt (Martin 2011). Reads were then processed in R using the DADA2 package (v1.32.0) (Callahan et al. 2016). First, forward reads were truncated at 250 bp and reverse reads at 200 bp according to their quality profiles and filtered using the parameters maxN = 0 and maxEE = c(2,2). Paired‐end reads were then merged, chimeras removed, and reads grouped into ASVs. Taxonomy was assigned to ASVs using the SILVA v138 database as a reference (Quast et al. 2013). The resulting ASV table and taxonomic assignment were combined with metadata using the phyloseq package (McMurdie and Holmes 2013). ASVs classified as chloroplast, mitochondria, or eukaryota were removed from the dataset. Samples with 1000 or fewer reads were discarded. After this filtering, 698 ASVs and 17 subsamples remained for analysis. Details of processing steps and read counts per sample are provided in Tables S1 and S2 (Supporting Information). Statistical analysis and visualisations were conducted using the microbiomeMarker and vegan packages (Cao et al. 2022; Oksanen et al. 2022). Each sample's percent relative abundance of each microbial phylum was calculated, and ASVs with a total relative abundance < 0.001% were removed. All additional analyses were conducted at the genus level. Plots were constructed using ggplot2 and tidyverse packages (Wickham et al. 2021, 2019). Alpha diversity was calculated using Shannon and Chao1 diversity indices. Beta diversity was estimated using the Aitchison distance, and patterns in microbial community composition were visualised using Principal Component Analysis (PCA). Heatmaps were generated using the Ampvis2 package (Andersen et al. 2018). To test for differences in beta diversity between groups, an analysis of similarities (ANOSIM) within the vegan package was used with 999 permutations. Analysis of Compositions of Microbiomes with Bias Correction (ANCOM‐BC) was used to find significantly enriched and differentially abundant taxa between depths.

## 3 Results

### 3.1 Seawater Temperature

Both depths recorded their lowest mean temperatures in March, with values of 12.79°C ± 0.43°C and 12.77°C ± 0.42°C at 10 m and 20 m, respectively (Figure 1a). Conversely, the highest mean temperatures occurred in August at 10 m (24.44°C ± 1.43°C) and in September at 20 m (22.29°C ± 1.27°C). In July and August, the mean temperature difference between the 10 m and 20 m depths was 1.63°C and 3.55°C, respectively. By September, the month of sample collection, this difference decreased to 0.7°C. Additionally, in August, the temperature exceeded 26°C on 9 days at 10 m and on 3 days at 20 m, highlighting the thermal variability between depths during the warmest period.

### 3.2 Microbiome

After quality filtering, 698 amplicon sequence variants (ASVs) were retained in the dataset, and 19 microbial phyla were identified. Bacterial communities from colonies at 13 m exhibited higher Shannon–Wiener alpha‐diversity than those at 17 m (Shannon index; *p* = 0.00099; Figure 1b). Beta diversity analysis revealed significant differences between the bryozoan microbiome of colonies collected at 13 and 17 m depths (ANOSIM; *p* = 0.019) (Figure 1c).

**FIGURE 2:**
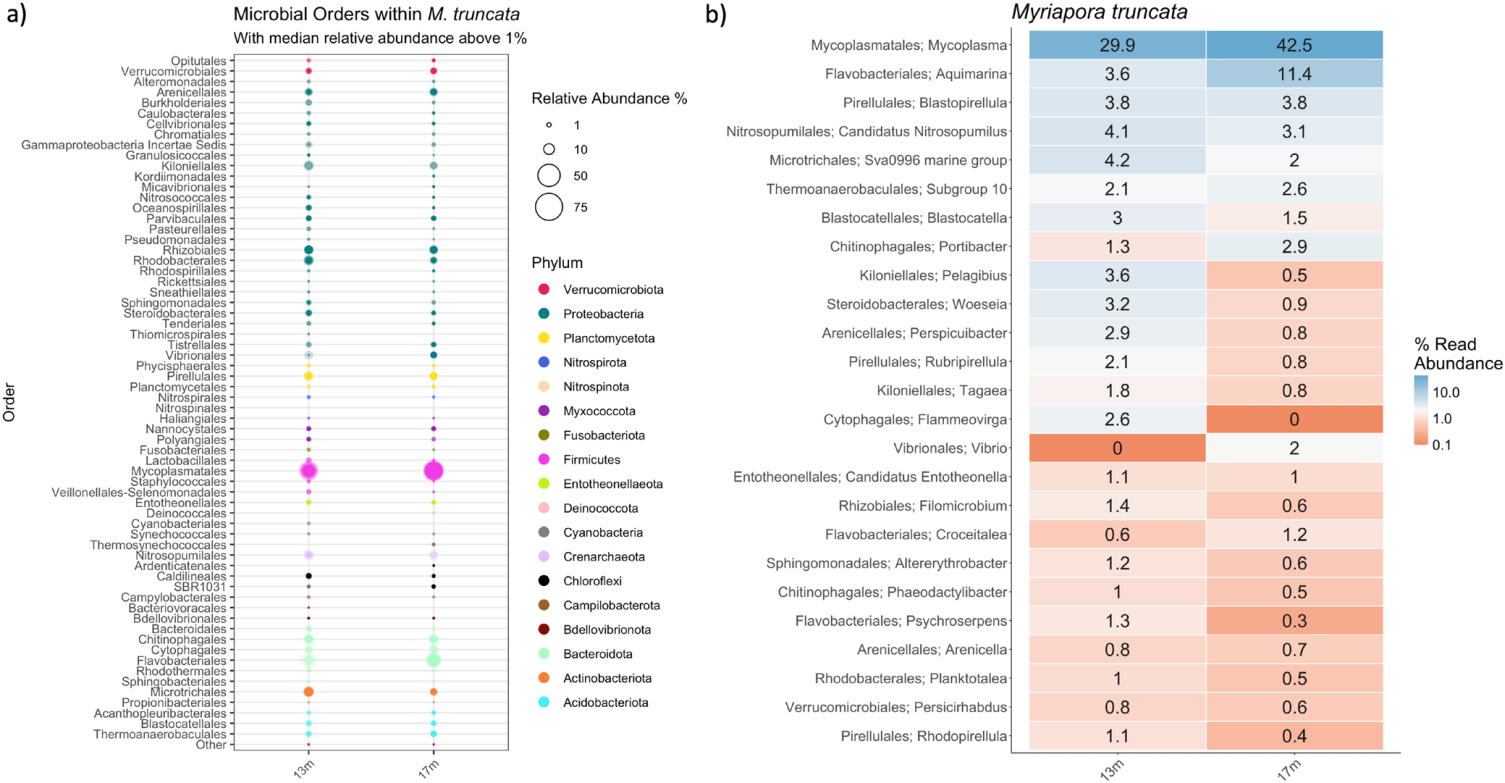
Relative abundance of the most abundant bacterial orders (a) and genera (b) in the microbiome of *Myriapora truncata* at the studied depths. In the bubble plot, circle sizes are proportional to the relative abundance, with colours indicating their respective phyla. Only taxa with a median relative abundance greater than 1% are shown; all others are grouped under ‘Other’. The outer, semi‐transparent bubble represents the upper bound of the standard deviation, while the solid inner circle denotes the lower bound. The heatmap represents relative abundance using a colour gradient, ranging from orange (low abundance) to dark blue (high abundance).

At the phylum level, the microbiome composition at both depths was found to be mainly composed of Proteobacteria, Bacteroidota, Firmicutes, and Planctomycetota (Figure 2a). Within the Proteobacteria, the microbiome from bryozoan colonies was found to be dominated by the orders Arenicellales, Rhizobiales, and Rhodobacterales. Abundant bacterial orders of the Bacteroidota included Chitinophagales, Cytophagales, and Flavobacteriales. Within the Firmicutes, the microbiome was found to be dominated by Mycoplasmatales. Within the Planctomycetota, Pirellulales was the dominant order (Figure 2a).

Colonies from both depths shared 11 abundant bacterial genera (*Mycoplasma*, *Aquimarina*, *Blastopirellula*, ‘*Candidatus Nitrosopumilus*’, *Sva0996 marine group*, *Subgroup 10*, *Blastocatella*, *Portibacter*, *Pelagibius*, *Woeseia* and *Perspicuibacter*) (Figure 2b).

The order Rhodobacteriales was dominant at both depths and showed higher relative abundance at 13 m, as illustrated by the heatmap of relative abundances (Figure 2a). However, differential abundance analysis using ANCOM‐BC did not identify any statistically significant differences for this order between the depths.

Similarly, the genera *Pelagibius*, *Woeseia*, *Perspicuibacter*, and *Rubripirellula* also showed increased abundance at this depth. In contrast, four dominant bacterial genera, *Aquimarina*, *Croceitalea*, *Mycoplasma*, and *Vibrio*, were depleted at the shallower depth (Figure 2b).

## 4 Discussion

This study represents the first characterisation of the microbiome of a bryozoan species after an extreme MHW event, providing new insights into its potential functional role under varying thermal conditions (Figure 1a). We identified five dominant bacterial orders—Rhizobiales, Rhodobacterales (Proteobacteria), Chitinophagales, Flavobacteriales (Bacteroidota) and Pirellulales (Planctomycetota)—which are commonly associated with *M. truncata* and other marine invertebrates, including corals (Figure 2a) (Figuerola et al. 2025; Gignoux‐Wolfsohn and Vollmer 2015; Lawler et al. 2016; McDevitt‐Irwin et al. 2017). This taxonomic overlap reflects a broadly conserved microbiome composition, consistent with the concept of a core microbiome in *M. truncata* that is shared across geographically distant Mediterranean sites (Figuerola et al. 2025).

Among these, 11 abundant bacterial genera—(such as *Aquimarina*, *Mycoplasma, Blastopirellula, Subgroup 10, Blastocatella*, *Portibacter* and *Perspicuibacter*)—were consistently detected across both depths and regions (Figure 2b), supporting their inclusion in the species' core microbiome. However, despite this overall compositional stability, we observed clear depth‐related differences in community diversity that appear linked to the intensity of thermal exposure during the 2022 MHW.

While Rhodobacterales were dominant at both depths, the heatmap indicated a higher relative abundance at 13 m compared to 17 m (Figure 2a), which may reflect sublethal stress, especially given the absence of visible necrosis. However, differential abundance analysis using ANCOM‐BC did not detect significant differences between depths, suggesting that changes in this order's abundance may not be strongly driven by depth‐related environmental factors. Although members of Rhodobacterales have been linked to thermal stress responses (Corinaldesi et al. 2022; Pootakham et al. 2018), these associations are highly context‐ and genus‐dependent. Further studies integrating functional profiling, host physiology, and temporal dynamics are needed to better understand the ecological role and health implications of Rhodobacterales shifts in bryozoan microbiomes.

The absence or extremely low abundance of *Endozoicomonas* (< 0.9%), particularly its complete absence in shallow colonies, is noteworthy (Figure 2b). This genus is widely recognised as a beneficial symbiont in tropical corals, involved in nutrient provision (sulfur, nitrogen and/or methane cycling and synthesis of amino acids) and secondary metabolites production (Morrow et al. 2015; Neave et al. 2016; van de Water et al. 2018). However, its ecological relevance in Mediterranean species, including bryozoans, remains poorly defined. In previous work, *Endozoicomonas* was detected in *M. truncata* at ~1.5% relative abundances under non‐stressful conditions (Figuerola et al. 2025). While the current decline is consistent with reductions reported under environmental stress such as climate anomalies in corals (McDevitt‐Irwin et al. 2017) and low pH conditions in *M. truncata* (Figuerola et al. 2025), its baseline presence and functional role in temperate systems may be inherently variable. Given the absence of pre‐heatwave data in this study and the fact that *Endozoicomonas* sometimes is rare or absent in some Mediterranean corals and other invertebrate microbiomes (Rubio‐Portillo et al. 2021), yet highly abundant in others (Bonacolta et al. 2024), we caution against interpreting its depletion as a definitive sign of holobiont health decline. Instead, we suggest it may serve as a candidate for further investigation aimed at identifying stress‐sensitive microbial indicators in this phylum.

Shallower colonies also exhibited increased microbial alpha diversity and a significantly different beta diversity compared to those at 17 m depth (Figure 1b,c). This suggests that these colonies are more impacted than those in deeper waters, likely due to the higher temperatures they are subjected (Figure 1a). This pattern is consistent with other studies on corals, where warming‐induced dysbiosis often results in increased diversity due to invasion by opportunistic bacteria (McDevitt‐Irwin et al. 2017). Such differences may serve as early warning signs of declining host health, as observed in Mediterranean gorgonians (Corinaldesi et al. 2022).

At finer taxonomic levels, several opportunistic genera—*Pelagibius*, *Woeseia*, *Perspicuibacter*, and *Rubripirellula*—were enriched at 13 m (Figure 2b), all previously linked to stressed corals or lobster shell disease (Feinman et al. 2017; Krishnaswamy et al. 2023; Quinn et al. 2012; Xu et al. 2023). In contrast, the bacterial genera considered part of the core microbiome of *M. truncata* (Figuerola et al. 2025) such as *Croceitalea*, along with *Aquimarina*, *Mycoplasma*, and *Vibrio*—identified as key symbionts in various temperate octocorals (van de Water et al. 2018)—were depleted at the shallower depth (Figure 2b). *Aquimarina*, in particular, is involved in nutrient acquisition and cycling and possesses genes associated with the production of antimicrobial compounds (Keller‐Costa et al. 2016). While *Mycoplasma* and *Vibrio* can include intracellular parasites and pathogenic species, respectively, they may also engage in mutualistic roles under stable conditions (van de Water et al. 2018), and their reduction here may reflect a broader shift in holobiont‐microbe homeostasis.

With MHWs expected to increase, understanding how marine holobionts can acclimate or resist repeated extreme events should be considered a conservation priority. Our findings reveal microbiome shifts in a common Mediterranean bryozoan species that may indicate reduced resilience to future MHWs. The dominance of opportunistic and potentially pathogenic bacteria across all colonies following an extreme MHW and their increased presence under warmer conditions suggest early signs of holobiont stress. Although *M. truncata* has shown some ability to withstand thermal stress (Pagès‐Escolà et al. 2018), and microbiomes may retain memory of past MHW exposures and acclimate under such conditions (Hackerott et al. 2021), extreme events like the 2022 MHW may pose a serious threat to this species. These results underscore the need for further research on marine invertebrate‐associated microbiomes, particularly in underrepresented phyla such as Bryozoa, to better assess the adaptive capacity of holobionts in a rapidly changing environment.

It is important to acknowledge some limitations in our study. Although the deeper colonies served as a comparative reference for lower thermal stress, the lack of physiological health markers makes it uncertain whether these deeper colonies truly represent unstressed or healthy conditions. Ideally, colonies collected from both depths prior to the summer period would provide a more robust baseline. Nonetheless, the overlap of core microbiome members detected here with those previously reported across Mediterranean populations (Figuerola et al. 2025) supports the biological relevance of our findings. Future studies that involve repeated sampling over time and physiological health indicators will be crucial for disentangling stress‐related microbial changes from natural variability for confirming the use of microbial taxa as reliable bioindicators of holobiont health.

## Author Contributions

Conceptualization: B.F. Methodology: B.F. and J.d.C. Investigation: B.F., C.L., C.A.E., P.L.S., J.G. and J.d.C. Formal analysis: B.F. Data curation: B.F. Project administration: B.F. Funding acquisition: B.F. and J.G. Writing – original draft: B.F. Writing – review and editing: B.F., C.L., C.A.E., P.L.S. and J.d.C.

## Ethics Statement

Fieldwork and sample collection were conducted under appropriate local permits and institutional guidelines. No protected or endangered species were harmed.

## Consent

The authors have nothing to report.

## Conflicts of Interest

The authors declare no conflicts of interest.

## Supporting information


**Figure S1:** Daily mean seawater temperature between 2018 and 2022 in Pota del Llop. Daily mean temperature was calculated from hourly measurements recorded by in situ hobo sensors at 10 and 20 m depth. Each panel corresponds to a different year, ordered from most recent (top) to oldest (bottom).
**Table S1:** Overview summary of processing steps. Initial ASVs were filtered to remove non‐bacterial reads (e.g., chloroplasts and mitochondria). Samples with < 1000 reads were excluded. Finally, rare ASVs were removed by retaining only those in the most abundant 99% of taxa across at least two samples.
**Table S2:** Number of sequencing reads retained per sample at key processing steps. Input refers to raw reads, Filtered to reads after quality filtering, and Non‐chimeric to final reads retained after chimera removal.

## Data Availability

The data that support the findings of this study are openly available in SRA at https://www.ncbi.nlm.nih.gov/bioproject/PRJNA1236962, reference number BioProject: PRJNA1236962.
