# Supplementary material for: Microbiome Composition in a Common Mediterranean Bryozoan Following an Unprecedented Marine Heatwave
Source: Environ Microbiol Rep. 2025 Sep 4;17(5):e70185. doi: 10.1111/1758-2229.70185 (PMC12409650; doi:10.1111/1758-2229.70185)
Supplement: Supplementary file 1 — Figure S1: Daily mean seawater temperature between 2018 and 2022 in Pota del Llop. Daily mean temperature was calculated from hourly measurements recorded by in situ hobo sensors at 10 and 20 m depth. Each panel corresponds to a different year, ordered from most recent (top) to oldest (bottom). Table S1: Overview summary of processing steps. Initial ASVs were filtered to remove non‐bacterial reads (e.g., chloroplasts and mitochondria). Samples with < 1000 reads were excluded. Finally, rare ASVs were removed by retaining only those in the most abundant 99% of taxa across at least two samples. Table S2: Number of sequencing reads retained per sample at key processing steps. Input refers to raw reads, Filtered to reads after quality filtering, and Non‐chimeric to final reads retained after chimera removal. [file EMI4-17-e70185-s001.docx]

**Supporting Information for**

**Microbiome composition in a common Mediterranean bryozoan following an unprecedented marine heatwave**

Blanca Figuerola^1*^, Cristina Linares^2,3^, Claudia Aparicio-Estalella^1^, Paula López-Sendino^1^, Joaquim Garrabou^1^, Javier del Campo^4^

^1^Departament de Biologia Marina i Oceanografia, Institut de Ciències del Mar (ICM-CSIC), Pg. Marítim de la Barceloneta 37-49, Barcelona 08003, Spain.

^2^Departament de Biologia Evolutiva, Ecologia i Ciències Ambientals, Facultat de Biologia, Universitat de Barcelona (UB), Barcelona 08028, Spain.

^3^Institut de Recerca de Biodiversitat (IRBio), Av. Diagonal, 643, 08028 Barcelona, Spain

^4^Institut de Biologia Evolutiva (CSIC– Universitat Pompeu Fabra), Pg. Marítim de la Barceloneta 37-49, 08003 Barcelona, Spain.

***Corresponding author**

**This PDF file includes:**

Figure S1

Tables S1-S2

**Fig. S1. Daily mean seawater temperature between 2018 and 2022 in Pota del Llop.** Daily mean temperature was calculated from hourly measurements recorded by in situ hobo sensors at 10 and 20 m depth. Each panel corresponds to a different year, ordered from most recent (top) to oldest (bottom). **
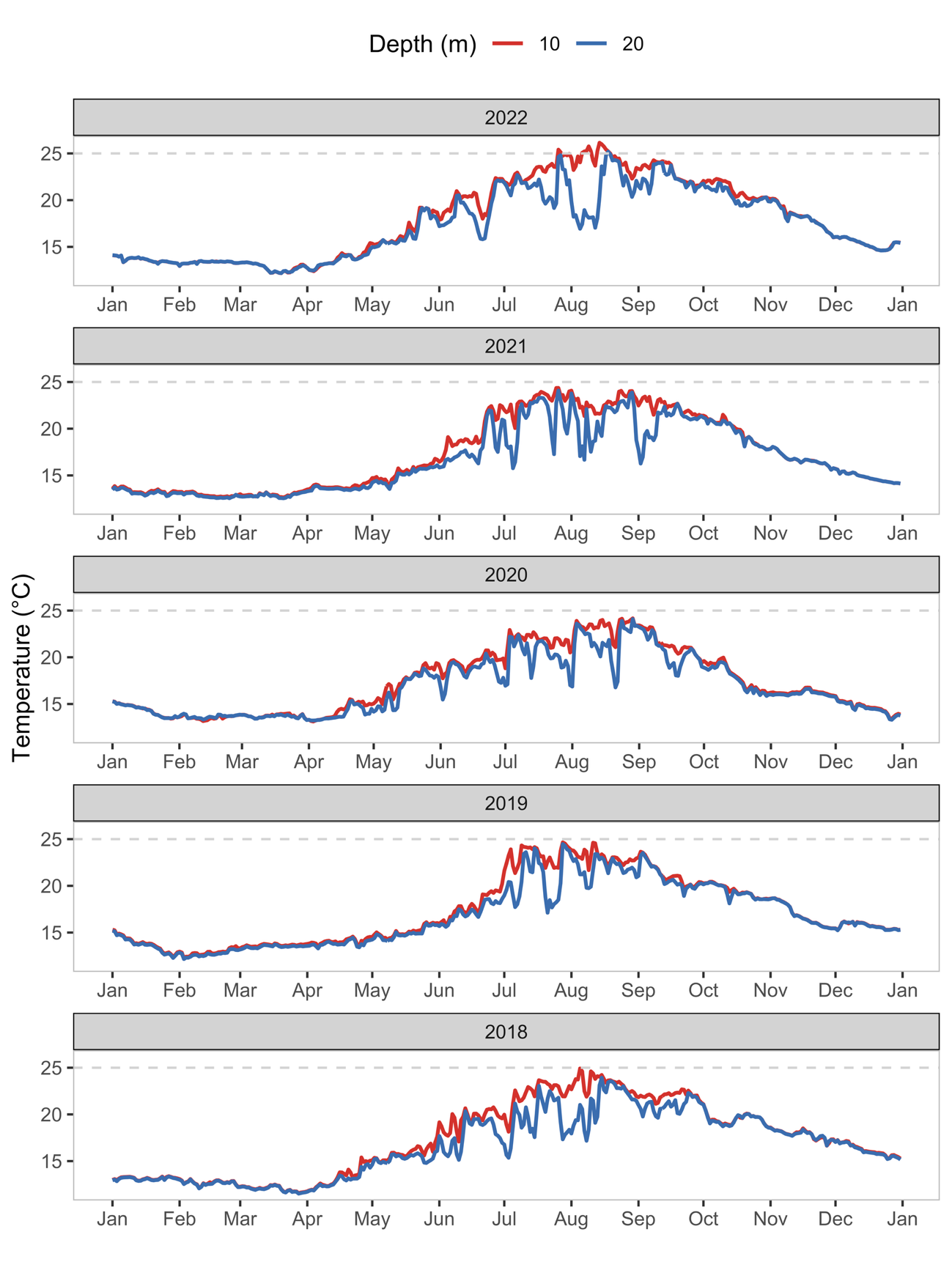
**

**Table S1. Overview summary of processing steps.** Initial ASVs were filtered to remove non-bacterial reads (e.g., chloroplasts and mitochondria). Samples with <1,000 reads were excluded. Finally, rare ASVs were removed by retaining only those in the most abundant 99% of taxa across at least two samples.

| **Processing Step** | **Samples** | **Taxa (ASVs)** |
| --- | --- | --- |
| Raw sequencing data | 20 | 3,460 |
| Removal of chloroplasts & mitochondria | 20 | 2,163 |
| Filtering low-depth samples (<1,000 reads) | 17 | 2,163 |
| Abundance filtering (top 99%) | 17 | 698 |

**Table S2.** Number of sequencing reads retained per sample at key processing steps. Input refers to raw reads, Filtered to reads after quality filtering, and Non-chimeric to final reads retained after chimera removal.

| **Sample ID** | **Colony** | **Depth (m)** | **Input** | **Filtered** | **Non-chimeric** |
| --- | --- | --- | --- | --- | --- |
| PL-M-13-1A | 1 | 13 | 18,115 | 16,834 | 15,656 |
| PL-M-13-1B | 1 | 13 | 25,315 | 23,391 | 21,466 |
| PL-M-13-1C | 1 | 13 | 284 | 238 | 138 |
| PL-M-13-2A | 2 | 13 | 6,412 | 5,960 | 5,455 |
| PL-M-13-2B | 2 | 13 | 8,690 | 7,975 | 7,214 |
| PL-M-13-2C | 2 | 13 | 6,572 | 6,022 | 5,457 |
| PL-M-13-3A | 3 | 13 | 37,289 | 34,518 | 31,701 |
| PL-M-13-3B | 3 | 13 | 34,546 | 32,201 | 30,145 |
| PL-M-13-3C | 3 | 13 | 11,238 | 10,478 | 9,795 |
| PL-M-17-1A | 1 | 17 | 33,752 | 31,384 | 29,844 |
| PL-M-17-1B | 1 | 17 | 42,282 | 39,519 | 37,385 |
| PL-M-17-1C | 1 | 17 | 29,140 | 27,260 | 25,985 |
| PL-M-17-2A | 2 | 17 | 26,300 | 24,484 | 22,552 |
| PL-M-17-2B | 2 | 17 | 4,088 | 3,768 | 3,121 |
| PL-M-17-2C | 2 | 17 | 10,671 | 9,897 | 9,059 |
| PL-M-17-3A | 3 | 17 | 28,623 | 26,583 | 25,060 |
| PL-M-17-3B | 3 | 17 | 15,012 | 14,093 | 13,361 |
| PL-M-17-3C | 3 | 17 | 31,074 | 29,062 | 27,492 |
